# Mutation in *NRAS* in familial Noonan syndrome – case report and review of the literature

**DOI:** 10.1186/s12881-015-0239-1

**Published:** 2015-10-14

**Authors:** Sara Ekvall, Maria Wilbe, Jovanna Dahlgren, Eric Legius, Arie van Haeringen, Otto Westphal, Göran Annerén, Marie-Louise Bondeson

**Affiliations:** Department of Immunology, Genetics and Pathology, Science for Life Laboratory, Uppsala University, Dag Hammarskjölds väg 20, 751 85 Uppsala, Sweden; Department of Paediatrics, the Sahlgrenska Academy, Gothenburg University, Gothenburg, Sweden; Department of Human Genetics, KU Leuven, Leuven, Belgium; Department of Clinical Genetics, Leiden University Medical Center, Leiden, The Netherlands

**Keywords:** *NRAS*, Noonan syndrome, Mutation, RAS-MAPK pathway, RASopathies

## Abstract

**Background:**

Noonan syndrome (NS), a heterogeneous developmental disorder associated with variable clinical expression including short stature, congenital heart defect, unusual pectus deformity and typical facial features, is caused by activating mutations in genes involved in the RAS-MAPK signaling pathway.

**Case presentation:**

Here, we present a clinical and molecular characterization of a small family with Noonan syndrome. Comprehensive mutation analysis of *NF1, PTPN11, SOS1, CBL, BRAF, RAF1, SHOC2, MAP2K2, MAP2K1, SPRED1, NRAS, HRAS* and *KRAS* was performed using targeted next-generation sequencing. The result revealed a recurrent mutation in *NRAS*, c.179G > A (p.G60E), in the index patient. This mutation was inherited from the index patient’s father, who also showed signs of NS.

**Conclusions:**

We describe clinical features in this family and review the literature for genotype-phenotype correlations for NS patients with mutations in *NRAS*. Neither of affected individuals in this family presented with juvenile myelomonocytic leukemia (JMML), which together with previously published results suggest that the risk for NS individuals with a germline *NRAS* mutation developing JMML is not different from the proportion seen in other NS cases. Interestingly, 50 % of NS individuals with an *NRAS* mutation (including our family) present with lentigines and/or Café-au-lait spots. This demonstrates a predisposition to hyperpigmented lesions in *NRAS-*positive NS individuals. In addition, the affected father in our family presented with a hearing deficit since birth, which together with lentigines are two characteristics of NS with multiple lentigines (previously LEOPARD syndrome), supporting the difficulties in diagnosing individuals with RASopathies correctly. The clinical and genetic heterogeneity observed in RASopathies is a challenge for genetic testing. However, next-generation sequencing technology, which allows screening of a large number of genes simultaneously, will facilitate an early and accurate diagnosis of patients with RASopathies.

## Background

Noonan syndrome (NS, OMIM 163950) is a relatively common developmental disorder belonging to the RASopathies, a group of clinically and genetically related syndromes [1, 2]. The molecular cause underlying RASopathies is dysregulation of the RAS-MAPK pathway and 15 different genes affecting this pathway have been associated to RASopathies. Of these 15 genes, eleven have been found to be involved in NS or NS-like conditions, where mutations in *PTPN11* are the cause of ~50 % of the cases. The other genes are *SOS1* [3, 4]*, CBL* [5–7]*, BRAF* [8]*, RAF1* [9, 10]*, SHOC2* [11]*, MAP2K1* [12]*, RIT1* [13]*, NRAS* [14], *KRAS* [15, 16] and *RRAS* [17]*.*

The main characteristics of NS are short stature, congenital heart defect, unusual pectus deformity and typical facial features, such as hypertelorism, ptosis, down-slanting palpebral fissures, low-set posteriorly rotated ears and a broad forehead. However, NS is a clinically variable disorder and additional associated features often present include neonatal failure to thrive, mild mental retardation, various skin manifestations, bleeding abnormalities and multiple skeletal defects [18, 19].

*NRAS* is a four-exon gene, encoding the widely expressed small GTPase NRAS, which act as a membrane-associated molecular switch in the RAS-MAPK pathway [14]. To date, only eight unrelated individuals with NS and three NS families have been identified with mutations in *NRAS* [14, 20–23].

Here, we performed a comprehensive molecular analysis of 13 RASopathy-associated genes; *NF1, PTPN11, SOS1, CBL, BRAF, RAF1, SHOC2, MAP2K2, MAP2K1, SPRED1, NRAS, HRAS* and *KRAS*, in a family with NS, which revealed a previously reported mutation in *NRAS*, c.179G > A (p.G60E). We describe clinical features in this family and review the literature for genotype-phenotype correlations for NS patients with mutations in *NRAS*.

## Case presentation

Clinical investigations and genetic analyses were performed according to the guidelines in the Declaration of Helsinki and approved by the ethical committee of Uppsala University and Gothenburg University, Sweden. Informed consent was obtained from all patients and specific permission was given for photographs.

### Case 1

**Fig. 1:**
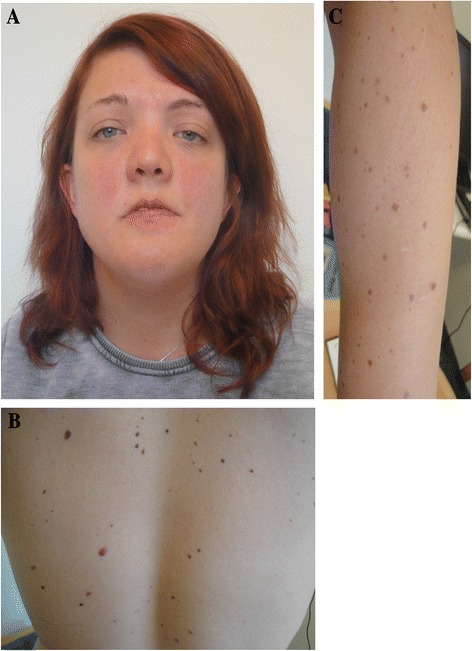
Photograph of the index patient affected by NS with a mutation in *NRAS*, p.G60E. **a** Facial features. **b** The back with multiple lentigines. **c** The left arm with multiple lentigines

This is a 28-year-old woman, who got the clinical diagnosis of Noonan syndrome (NS) at the age of 4 years because of growth retardation, cardiomyopathy and facial features. She is the only child of non-related parents. The father (Case 2) has facial features of NS, but few additional clinical symptoms. She was born to a mother with diabetes during pregnancy with a birth weight of 4.7 kg (+3 SDS), a length of 52 cm (+1 SDS) and a head circumference of +2 SDS. She also had a large left ventricle, and a systolic murmur, but this disappeared at the age of six years. Postnatally, her growth decelerated and she had feeding difficulties. At 6.5 years of age, her height was 104 cm (−2 SDS) and her weight 18.5 kg (−2 SDS). She had low endogenous growth hormone (GH) secretion defined as “partial GH deficiency”, and started GH therapy within a formal clinical trial (NovoNordisk) from 6.5 years of age. She was treated with GH (dose of 66 μg/kg/day) and responded exceptionally well and treatment was discontinued after two years. However, at 10 years of age, she had her first pubertal signs and GH-treatment was started again using a standard dose of 33 μg/kg/day. At 12.3 years of age, she had menarche. The GH-treatment continued until final height (FH) was reached at the age of 14 years. Her FH is 164.5 cm (−0.45 SDS) and weight of 60 kg (+0.3 SDS). Her psychomotor development is normal, but she has slight problems of attention deficit. She attended regular school and works as an assistant nurse. At the age of 24 years, she has the following features of NS (Fig. 1a): a large skull (62 cm) with a broad forehead, hypertelorism, down slanted palpebral fissures, bilateral ptosis (especially of her left eye), short and broad neck with a low hairline, and low-set ears with broad helices. Her hair is normal. She has two large Café-au-lait spots on her back and >50 freckles (lentigines) all over her body, especially on her back (Fig. 1b) and arms (Fig. 1c).

### Case 2

**Fig. 2:**
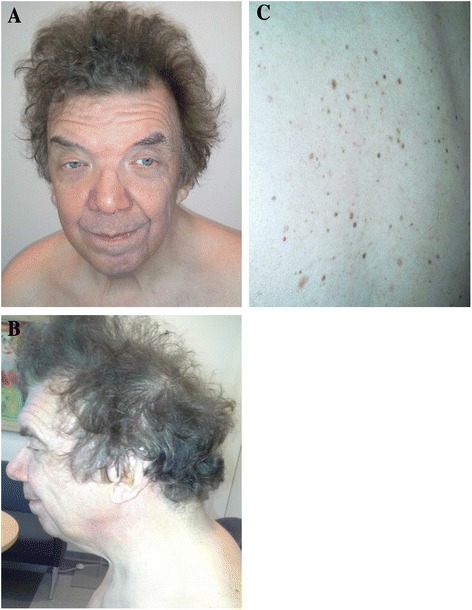
Photograph of the affected father with the same *NRAS* mutation, p.G60E. **a** Frontal facial features. **b** Additional facial features. **c** The back with multiple lentigines

This is the 62-year-old father of Case 1. He was clinically diagnosed after Case 1 was diagnosed. He has facial features of NS, but few additional clinical symptoms. Sensorineural hearing impairment was present at birth. His growth pattern was normal, but he had a delayed puberty. His FH is 175.0 cm (−0.4 SDS) and weight 75 kg (±0 SDS). The intellectual development was normal. He followed normal school and university education and worked as a librarian until the age of 55 years, when he had to retire because of tinnitus. At the age of 62 years, he has the following features of NS (Fig. 2a–c): slight macrocephaly (61 cm, +2 SDS), bilateral ptosis, hypertelorism and down-slanting palpebral fissures. He also has curly hair and lentigines on his back. He had a cardiac murmur in childhood that disappeared spontaneously.

### Methods

Genomic DNA from the index patient and her father was extracted from peripheral blood leukocytes according to standard procedures.

#### Mutation analysis

The index patient was analyzed for variants in all coding exons and exon-intron boundaries of *NF1* (NM_000267.3), *PTPN11* (NM_002834), *SOS1* (NM_005633), *CBL* (NM_005188), *BRAF* (NM_004333), *RAF1* (NM_002880), *SHOC2* (NM_007373), *MAP2K2* (NM_030662), *MAP2K1* (NM_002755), *SPRED1* (NM_152594)*, NRAS* (NM_002524), *HRAS* (NM_005343) and *KRAS* (NM_004985) using Agilent HaloPlex Target Enrichment (Agilent Technologies, Inc., Santa Clara, CA, USA), followed by next-generation sequencing on MiSeq, Illumina (Illumina, Inc., San Diego, CA, USA). Data analysis was performed by NextGENe software v2.3.1 (SoftGenetics, LLC., State College, PA, USA) and BENCHlab NGS (Cartagenia, Inc., Cambridge, MA, USA) [Ekvall et al. Manuscript in preparation].

Variants observed in *NRAS* exon 2 were verified by bi-directional Sanger sequencing in the index patient and her father. Primer sequences and PCR conditions are available upon request.

### Results

DNA sequencing analysis of the index patient (Fig. 1) was performed on 13 RASopathy-associated genes using HaloPlex target enrichment (Agilent) and next-generation sequencing on MiSeq (Illumina). Coverage and read depth of the RASopathy genes in the index patient is shown in Table 1. Targeted bases in region of interest (ROI) with >30X read depth was 100 % for all genes, except *NF1* (99.8 %) and *SOS1* (99.9 %). No complementary Sanger sequencing was performed. A heterozygous missense mutation, c.179G > A; p.G60E, in exon 2 of *NRAS* was identified and verified using Sanger sequencing. This mutation was inherited from the father (Fig. 2), who also shows signs of NS. No additional variants of significance were identified in the index patient.

**Table 1:** Average read depth and coverage of RASopathy-associated genes in this study

Gene	Reference sequence	ROI^a^ bases	Exons	Average read depth	Coverage >30X (%)
*BRAF*	NM_004333	3021	18	12 637	100. 0
*CBL*	NM_005188	3361	16	12 016	100.0
*HRAS*	NM_005343	812	6	14 291	100.0
*KRAS*	NM_004985	768	5	10 811	100.0
*MAP2K1*	NM_002755	1622	11	12 525	100.0
*MAP2K2*	NM_030662	1643	11	11 127	100.0
*NF1*	NM_000267	10737	58	12 728	99.8
*NRAS*	NM_002524	861	7	16 378	100.0
*PTPN11*	NM_002834	6521	16	16 262	100.0
*RAF1*	NM_002880	2587	16	15 985	100.0
*SHOC2*	NM_007373	2069	8	17 812	100.0
*SOS1*	NM_005633	4922	23	13 439	99.9
*SPRED1*	NM_152594	1615	7	10 509	100.0

^a^ *ROI* Region of interest

## Conclusions

To date, only eight unrelated patients with NS and three NS families have been reported positive for *NRAS* mutations (p.G13D, p.I24N, p.P24L, p.T50I and p.G60E) [14, 20–23]. In this study, we describe an additional family with NS, where the index patient and her father have c.179G > A (p.G60E). This mutation has been identified in both sporadic and familial NS patients of European origin [14, 23] and is the most common germline mutation in *NRAS*.

NS patients with *NRAS* mutations often show a relatively mild phenotype of typical Noonan facial features. A comparison of previously reported *NRAS-*associated NS cases shows that all of the patients present with typical Noonan facial features (14/14) and 11/13 have macrocephaly or relative macrocephaly, but only half of them display congenital heart defects (7/14). All previously reported patients also show short stature. However, in the family reported here the father’s height was normal, while the daughter had short stature successfully treated with GH. The majority has pterygium or webbing of the neck (10/12). Thorax deformity (pectus excavatum) occurs in 5/14 patients, while easy bruising is less common (3/14). Half of the males show cryptorchidism (6/10) and ophthalmological problems appear in 4/14 patients. Motor delay is common (9/14 patients) and as previously reported, intellectual development is often mildly delayed (6 patients normal and 8 mildly delayed). Keratosis pilaris/hyperkeratosis is less common (4/12 patients) and hair abnormalities occur in about half of the patients. Of note, lentigines are observed in six patients, but leukemia/cancer are rarely seen (1 patient with JMML) (Table 2) [14, 20–23]. Somatic mutations affecting genes in the RAS-MAPK pathway are associated with cancer, and NS and related disorders are known to cause a predisposition to cancer [24]. Somatic mutations in *NRAS* are involved in the development of hematological malignancies and in a variety of solid tumors (COSMIC database; http://cancer.sanger.ac.uk/). However, germline *NRAS* mutations differ from most common somatic *NRAS* mutations associated with malignancies and are less activating in dysregulating intracellular signaling [18].

**Table 2:** Clinical features of patients with Noonan syndrome caused by *NRAS* mutations

#	1	2	3	4	5	6	7	8	9	9 M	10	11	12	12 F
Patient	De Filippi et al. [20]	Runtuwene et al. [21]	Denayer et al. [22]	Denayer et al. [22]	Denayer et al. [22]	Cirstea et al. [14]	Cirstea et al. [14]	Cirstea et al. [14]	Cirstea et al. [14]	Cirstea et al. [14]	Kraoua et al. [23]	Kraoua et al. [23]	Present study	Present study
*NRAS* mutation	p.G13D	p.I24N	p.I24N	p.P24L	p.T50I	p.T50I	p.T50I	p.G60E	p.G60E	p.G60E	p.G60E	p.G60E	p.G60E	p.G60E
Origin of mutation	*de novo*	*de novo*	*de novo*	Inherited	ND	*de novo*	*de novo*	*de novo*	Inherited	ND	ND (probably inherited)	*de novo*	Inherited	ND (probably inherited)
Paternal age at conception	ND	26 years	ND	ND	ND	50 years	34 years	31 years	47 years	44 years	45 years	47 years	34 years	ND
Age at last examination	3 years	30 years	13 years	19 years	2.5 years	14 years	7 years	3.3 years	20 years	50 years	24 years	3 months	28 years	62 years
Gender	Male	Male	Male	Male	Male	Male	Male	Female	Male	Female	Male	Female	Female	Male
Prenatal findings	ND	Polyhydramnios	ND	ND	ND	Nuchal edema, Polyhydramnios	Polyhydramnios	Single umbilical artery	-	-	Polyhydramnios	Pyelectasis	-	-
Congenital heart defect	-	-	-	ND	Coarctation aortae, Patent foramen ovale	HCM	PS	Mild HCM, Mitral valve dysplasia, PS	-	-	-	PS	ASD, HCM	Cardiac murmur
Rythm disturbance	ND	-	ND	ND	-	SVES	-	-	-	-	-	-	-	-
Typical facial features	+	+	+	+	+	+	+	+	+	+	+	+	+	+
Stature	5–10^th^ centile	Mild short	<3^rd^ centile	10^th^–25^th^ centile	10^th^–25^th^ centile	10^th^ centile^a^	<3^rd^ centile	<3^rd^ centile	>10^th^ centile	10^th^ centile	3^rd^ centile	3^rd^–10^th^ centile	50^th^ centile^a^	50^th^ centile
Macrocephaly	Relative	>90^th^ centile	>97^th^ centile	ND	25^th^–50^th^ centile	+	Relative	-	+	+	Relative	Relative	+	Relative
Pterygium colli/Webbed neck	-	+	ND	ND	+	+	-	+	+	+	+	+	+	+
Thorax deformity	-	Pectus excavatum	Pectus excavatum	ND	Pectus excavatum	+	-	Pectus excavatum	+	+	Mildly depressed thorax	Pectus excavatum	-	-
Easy bruising	-	-	ND	+	ND	-	-	-	-	-	+	ND	+	-
Cryptorchidism	-	+	+	ND	+	+	+	NA	+	NA	-	NA	NA	-
Ophthalmological problems	ND	-	Strabismus, Bilateral keratoconus of the cornea	ND	ND	Myopia	-	-	-	Myopia	-	-	Astigmatis, Myopia, Strabismus	-
Motor delay/Muscular hypotonia	-	Motor delay	Mild	ND	ND	Mild	+	+	+	+	Mild	+	-	-
Mental development	Normal	Mild learning difficulties	Normal	Learning difficulties	Normal	Normal	Borderline	Speech delay	Normal-borderline	Normal	Speech delay, dyscalculy	NA	ADHD, normal IQ	Normal
Keratosis pilaris/Hyperkeratosis	ND	-	ND	ND	ND	Severe	-	+	+	+	ND	-	-	-
Hair abnormalities	-	-	ND	ND	ND	Curly hair	Curly hair	Sparse thin hair	Curly hair	-	-	Curly hair	-	Curly hair
Lentigines/Café-au-lait spots	+	Some lentigines	-	-	-	-	-	-	-	-	+	+	+	+
Leukemia/Cancer	JMML	-	-	-	-	-	-	-	-	-	-	-	-	-
Other	-	Oligospermia	-	Inadequate visio-spatial orientation skills, Inguinal hernia, Delayed pubertal development	-	Pes equinovarus	-	Palpebral ptosis	Ichtyosiform eczema, Acanthosis nigricans, Scoliosis	Mother of patient 9	Palpebral ptosis, Inguinal hernia, Scoliosis	Palpebral ptosis, Unilateral pyelectasis	-	Sensory-neural hearing deficit, Father of patient 12

*ASD* atrial septal defect, *HCM* hypertrophic cardiomyopathy, *JMML* juvenile myelomonocytic leukemia, *NA* not applicable, *ND* not determined, *PS* pulmonic stenosis, *SVES* supraventricular extrasystole

^a^Received growth hormone treatment from the age of 8 years, when partial growth hormone deficiency had been noted

In summary, we report an NS family with a p.G60E in *NRAS*. Neither of affected individuals presented with JMML. Thus, the proportion of JMML observed in *NRAS* patients (1/12) is comparable with the observed proportion of JMML in NS in general [25].

Interestingly, half of the patients (including affected individuals in our family) presented with lentigines and/or Café-au-lait spots, which is high compared to the prevalence of 3 % for lentigines and 10 % for Café-au-lait spots in the general NS population [26]. Multiple nevi, lentigines and/or Café-au-lait spots are also detectable in one-third of NS individuals with a *RAF1* mutation and previous studies have demonstrated a higher prevalence of these features in *BRAF*-positive NS individuals as well. This suggests that NS individuals with a mutation in *NRAS, RAF1* or *BRAF* have a predisposition to hyperpigmented cutaneous lesions [8, 27]. Of note, the father in our family presented with congenital sensorineural hearing impairment, which together with lentigines are two common features in Noonan syndrome with multiple lentigines (NS-ML, previously LEOPARD syndrome). This demonstrates the wide spectrum of phenotypes within each syndrome as well as the clinical overlap between RASopathies, which makes diagnosis of NS and related disorders challenging.

However, by using the advent of next-generation sequencing technology, which allow for screening of a large number of genes simultaneously, an early and accurate genetic diagnosis of patients with RASopathies will be facilitated.

## Consent

We have obtained written informed consent from the patients for publication of this case report and accompanying images. A copy of the written consent is available from the Editor of this journal.

## Abbreviations

NS: Noonan syndrome
MAPK: Mitogen-activated protein kinase
JMML: Juvenile myelomonocytic leukemia
GH: Growth hormone
ROI: Region of interest
COSMIC: Catalogue of somatic mutations in cancer
ASD: Atrial septal defect
HCM: Hypertrophic cardiomyopathy
NA: Not applicable
ND: Not determined
PS: Pulmonic stenosis
SVES: Supraventricular extrasystole
